# Antioxidant Activities and Repair Effects on Oxidatively Damaged HK-2 Cells of Tea Polysaccharides with Different Molecular Weights

**DOI:** 10.1155/2018/5297539

**Published:** 2018-11-21

**Authors:** Xin-Yuan Sun, Jian-Min Wang, Jian-Ming Ouyang, Li Kuang

**Affiliations:** Institute of Biomineralization and Lithiasis Research, Jinan University, Guangzhou 510632, China

## Abstract

This study aims at investigating the antioxidant activity and repair effect of green tea polysaccharide (TPS) with different molecular weights (Mw) on damaged human kidney proximal tubular epithelial cells (HK-2). Scavenging activities on hydroxyl radical (^·^OH) and ABTS radical and reducing power of four kinds of TPS with Mw of 10.88 (TPS0), 8.16 (TPS1), 4.82 (TPS2), and 2.31 kDa (TPS3) were detected. A damaged cell model was established using 2.6 mmol/L oxalate to injure HK-2 cells. Then, different concentrations of TPSs were used to repair the damaged cells. Index changes of subcellular organelles of HK-2 cells were detected before and after repair. The four kinds of TPSs possessed radical scavenging activity and reducing power, wherein TPS2 with moderate Mw presented the strongest antioxidant activity. After repair by TPSs, cell morphology of damaged HK-2 cells was gradually restored to normal conditions. Reactive oxygen species production decreased, and mitochondrial membrane potential (Δ*ψ*m) of repaired cells increased. Cells of G1 phase arrest were inhibited, and cell proportion in the S phase increased. Lysosome integrity improved, and cell apoptotic rates significantly reduced in the repaired group. The four kinds of TPSs with varying Mw displayed antioxidant activity and repair effect on the mitochondria, lysosomes, and intracellular DNA. TPS2, with moderate Mw, showed the strongest antioxidant activity and repair effect; it may become a potential drug for prevention and treatment of kidney stones.

## 1. Introduction

Tea originated from China features a long history of over 4000 years; it is also the most popular nonalcoholic beverage and common food ingredient in Asia [1]. Tea polysaccharide (TPS) is an acid polysaccharide extracted from tea leaves [2]. TPS displays antioxidant, hypoglycemic, hypolipidemic, antihypertensive, immunological, antitumor, anticoagulant, and protective effects [3]. Chen et al. [4] have confirmed that TPS isolated from green tea manifests the scavenging activity of superoxide radicals, hydroxyl radicals (^·^OH), and lipid radicals in vitro. TPS exerts protective effects against cellular damage induced by oxidative stress.

Many studies have shown that tea polyphenol has inhibitory effects on calcium oxalate urolithiasis due to its antioxidative effects [5, 6]. Tea polyphenol decreases osteopontin expression and cell apoptosis and increases superoxide dismutase activity in rat kidney tissues, thus inhibits the formation of calcium oxalate stones [5]. Polyphenols mainly act as antioxidants through phenolic hydroxyl groups. By contrast, TPSs contain not only hydroxyl groups but also more carboxyl groups. The binding ability of carboxyl groups to calcium ions is obviously higher than that of hydroxyl groups [7], so it has a better potential to inhibit the formation of stones. At the same time, the structure of plant polysaccharides is similar to glycosaminoglycans (GAG), which are potent inhibitors of growth and aggregation of calcium oxalate crystals in vitro [8]. Research has shown that semisynthetic polysaccharides are more effective in preventing crystal-cell interactions than are GAGs [9].

The molecular weight (Mw) and structure of TPS are related to tea species and purification [10, 11]. Chen et al. [12] revealed that the TPS extracted from green tea, with Mw of 120 kDa, comprises arabinose, ribose, xylose, glucose, galactose, and uronic acid at a molar ratio of 1.00 : 0.77 : 2.65 : 0.88 : 0.42 : 2.13. Wang et al. [13] extracted from green tea a water-soluble polysaccharide (7WA), with an average Mw of 7.1 × 10^4^ Da. 7WA mainly contains arabinose and galactose at a molar ratio of 1.0 : 0.96 and possesses a backbone consisting of 1,3- and 1,6-linked galactopyranosyl residues, with branches attached to O-3 of 1,6-linked galactose residues and O-4 and O-6 of 1,3-linked galactose residues. Wang et al. [14] achieved a polysaccharide component (ZTPs) from green tea with a Mw of 8000 Da by hot-water extraction and ethanol precipitation. ZTPs consist of mannose, ribose, rhamnose, glucuronic acid, galacturonic acid, glucose, xylose, galactose, arabinose, and fucose, with molar percentages of 4.3%, 1.4%, 4.1%, 2.6%, 3.0%, 31.4%, 4.6%, 21.8%, 23.5%, and 3.3%, respectively.

Polysaccharide bioactivity is closely related with molecular structure, including Mw, active group content, structure of the main chain and branched chain, monosaccharide composition and sequence, glycosidic bond type and position, conformation, and solubility [15–17]. For the same kind of polysaccharides, Mw is the most important indicator of biological activity [18–21]. Lei et al. [19] showed that three sulfated glucans from *Saccharomyces cerevisiae*, with Mw of 12.9 (sGSC1), 16.5 (sGSC2), and 19.2 kDa (sGSC3), displayed antioxidant and immunological activities in vitro. Results showed that sGSC1, sGSC2, and sGSC3 can scavenge 1,1-diphenyl-2-picryl-hydrazyl (DPPH), superoxides, and hydroxyl radicals, and strength of radical scavenging effects of sGSCs followed the order sGSC1 > sGSC2 > sGSC3. Sun et al. [20] performed degradation of *Porphyridium cruentum* (EPS-0) with Mw of 2918.7 kDa to obtain three polysaccharide fractions with low Mw of 256.2 (EPS-1), 60.66 (EPS-2), and 6.55 kDa (EPS-3). EPS-0 showed no remarkable antioxidant activity, but polysaccharide fractions after degradation exerted inhibitory effects on hemolysis injury induced by Fe^2+^/Vc in mouse liver hemocytes; half maximal inhibitory concentration (IC_50_) value of EPS-1, EPS-2, and EPS-3 measured 1.09, 0.91, and 0.81 mg/mL, respectively. Results suggested that EPS-3, with the lowest Mw, showed the strongest protective effect on oxidative damage of liver hemocytes in mice. Ying et al. [21] extracted and obtained three Liubao TPS sections with Mw of 7.1 kDa (LTPS-30), 6.9 kDa (LTPS-50), and 6.6 kDa (LTPS-70). LTPS-70, with the smallest Mw, exhibited the strongest antioxidant activity and repair effect on damaged human umbilical vascular endothelial cells in the concentration range of 12.5–400 *μ*g/mL.

Oxidative injury is one of the main factors that cause various diseases. Formation of kidney stones is related to oxidative damage of kidney epithelial cells [22–24]. TPS presents good antioxidant activity, which can reduce oxidative damage and can repair cells [25]. Therefore, TPS may be used to reduce incidences and prevent formation of kidney stones. We extracted four kinds of green TPSs with Mw of 10.88, 8.16, 4.82, and 2.31 kDa and comparatively investigated their antioxidant activity and repair effect on damaged kidney epithelial cells to provide basis for prevention and treatment of kidney stones.

## 2. Materials and Methods

### 2.1. Reagents and Apparatus

Green tea polysaccharide (TPS) was purchased from Shaanxi Ciyuan Biological Co., Ltd.; D_2_O (99.9%, Sigma), other conventional reagents were purchased from Guangzhou Chemical Reagent Company (Guangzhou, China).

Human kidney proximal tubular epithelial (HK-2) cells were purchased from Shanghai Cell Bank, Chinese Academy of Sciences (Shanghai, China). Fetal bovine serum and cell culture medium (DMEM) were purchased from HyClone Biochemical Products Co. Ltd. (Beijing, China). Cell culture plates of 6-, 12-, and 96-well (NEST, China). Cell proliferation assay kit (Cell Counting Kit-8, CCK-8) was purchased from Dojindo Laboratory (Kumamoto, Japan). Acridine orange (AO) was purchased from Siama (USA). Hematoxylin and eosin (HE) staining kit, reactive oxygen species assay kit (DCFH-DA), and mitochondrial membrane potential assay kit (JC-1) were purchased from Shanghai Beyotime Bio-Tech Co., Ltd. (Shanghai, China). Carbonyl cyanide 3-chlorophenylhydrazone (CCCP) and cell apoptosis and necrosis assay kit were purchased from 4A Biotech Co., Ltd. (Beijing, China).

The apparatus included ultraviolet-visible spectrophotometer (Cary 500, Varian company, USA), inverted fluorescence microscope (Olympus company, Japan), flow cytometry (Beckman, Gallios, USA), enzyme mark instrument (safireZ, Tecan, Switzerland), nuclear magnetic resonance spectrometer (Varian Bruker-300 MHz, Germany), and Fourier-transform IR spectra (FT-IR) (EQUINOX55, Bruker, Germany).

### 2.2. Degradation of Tea Polysaccharide

About 1.2 g of crude tea polysaccharide (TPS0) was weighted accurately and dissolved in 20 mL distilled water. The reaction system was quickly added with hydrogen peroxide (H_2_O_2_) and allowed to proceed for 2 h at 90°C; at which point, the solution PH was adjusted to 7.0 by adding 2 mol/L NaOH solution. The degraded solution was concentrated to one-third of its original volume at 60°C. The product was precipitated by adding anhydrous ethanol three times. The solution was stored overnight and filtered. The filtrate was dried in vacuum to obtain the degraded polysaccharide. Degraded tea polysaccharides with different molecular weight can be gained by changing the concentration of H_2_O_2_ at 4%, 8%, and 14%, respectively.

### 2.3. Molecular Weight Determination of Tea Polysaccharide

According to reference [26], the Ubbelohde viscosity was used to measure the molecular weight of tea polysaccharide. The intrinsic viscosity [*η*] and molecular weight M could be described by the Mark-Houwink empirical equation [*η*] = *κ*M^*α*^. For tea polysaccharide, the *κ* and *α* are 0.0416 and 0.49, respectively.

### 2.4. Analysis of Carboxylic Group Content of Tea Polysaccharide

The carboxylic group (-COOH) content of TPS was measured by conductometric titration [27]. The final value was the average of three parallel experiments.

### 2.5. Fourier-Transform Infrared Spectroscopy (FT-IR) Analysis of Tea Polysaccharide

The dried polysaccharide sample (2.0 mg each) was mixed with 200 mg of potassium bromide (KBr) and compressed for scanning the spectrum in the region of 4000 cm^−1^ to 400 cm^−1^ with a resolution of 4 cm^−1^.

### 2.6. ^1^H NMR and ^13^C NMR Spectrum of Tea Polysaccharide

According to reference [28], approximately 40 mg of tea polysaccharide was dissolved in 0.5 mL deuterium oxide (D_2_O, 99.9%) in NMR tube. After the polysaccharide was dissolved completely, the ^1^H and ^13^C NMR spectrum was performed using the Varian Bruker-600 MHz spectrophotometer.

### 2.7. Hydroxyl Radical (^·^OH) Scavenging Activity of TPS with Different Molecular Weight

The ^·^OH scavenging ability of polysaccharide in vitro was detected by H_2_O_2_/Fe system method [19, 29]. 38 EP tubes (10 mL) were prepared, and the reaction mixture in the EP tube that contained different concentrations of polysaccharides (0.15, 0.5, 0.8, 1.0, 2.0, and 3.0 g/L) was incubated with FeSO_4_ (2.5 mmol/L, 1 mL) and phenanthroline (2.5 mmol/L, 1 mL) in a phosphate buffer (20 mmol/L, 1 mL, pH 6.6) for 90 min at 37°C. The absorbance measured at 580 nm repeatedly took average value. The ascorbic acid (Vc) was used as a positive control group. The ability to scavenge hydroxyl radicals was calculated using the following equation:

(1) $$\begin{array}{c} Scavenging\;effect\;\left(\%\right)=\frac{\left(A_{3}-A_{1}\right)}{\left(A_{2}-A_{1}\right)}\times 100, \end{array}$$

where *A*_1_ is the blank group; *A*_2_ is the control group with H_2_O_2_; and *A*_3_ is the experiment group with polysaccharide.

### 2.8. ABTS Radical Scavenging Activity of TPS with Different Molecular Weight

The ABTS radical scavenging activity of polysaccharides was performed according to [30] with slight modification. 7 mmol/L ABTS solution was mixed with 2.45 mmol/L potassium persulfate aqueous solution, and then, the mixture was incubated in the dark at room temperature for 14 h. Then, 3.0 mL mixture solution was added to 1 mL of various polysaccharide solutions in a test tube. After reacting for 6 min at room temperature, the absorbance was measured at 734 nm.

(2) $$\begin{array}{c} Scavenging\;effect\;\left(\%\right)=\left[1-\frac{\left(A_{1}-A_{2}\right)}{A_{0}}\right]\times 100, \end{array}$$

where *A*_0_ is the control group without polysaccharide; *A*_1_ is the experiment group; and *A*_2_ is the blank group without reagents.

### 2.9. Reducing Power of TPS with Different Molecular Weight

The reducing power of polysaccharides was determined referring to reference [31] with some modifications. 2 mL of four polysaccharide samples with different molecular weights in different concentrations (0.15, 0.5, 0.8, 1.0, 2.0, and 3.0 g/L) was mixed with 2 mL phosphate buffer (PBS, pH = 6.6) and 2 mL potassium ferricyanide (1.0%, *w*/*v*). The mixture was incubated at 50°C for 20 min and cooled to room temperature. 2 mL trichloroacetic acid (10%, *w*/*v*) was added to the mixture which was then centrifuged for 10 min at 3000 r/min. The supernatant (2 mL) was mixed with 0.5 mL FeCl_3_ (0.1%, *w*/*v*) solution and 2 mL distilled water. The mixture was fully mixed and stood for 10 min. The absorbance was measured at 700 nm. The phosphate buffer was used as a negative control group. The ascorbic acid (Vc) was used as a positive control group and for comparison.

### 2.10. Cytotoxicity Measurement of TPS on HK-2 Cells

HK-2 cells were cultured in DMEM-F12 medium containing 10% fetal bovine serum, 100 *μ*g/mL streptomycin antibiotic-100 U/mL penicillin with pH 7.4 in a 5% CO_2_ humidified environment at 37°C. Upon reaching a monolayer of 80%–90% confluence, cells were gently blown after trypsinization to form a cell suspension for subsequent cell experiments.

Cell suspension with a cell concentration of 1 × 10^5^ cells/mL was inoculated per well in 96-well plates and incubated for 24 h. Afterward, the culture medium was removed, and 100 *μ*L of 0, 20, 60, and 100 *μ*g/mL TPSs with various molecular weights was added and each concentration was repeated in three parallel wells. After incubation for 24 h, 10 *μ*L CCK-8 was added to each well and incubated for 1.5 h. Absorbance (A) was measured by using the enzyme mark instrument at 450 nm according to the CCK-8 kit instruction. Cell viability was determined using the following equation:

(3) $$\begin{array}{c} Cell\;viability\left(\%\right)=\frac{A\;\left(treatment\;group\right)}{A\;\left(control\;group\right)}\times 100. \end{array}$$

### 2.11. Repair Effect of TPS on Damaged HK-2 Cells by CCK-8

100 *μ*L of cells suspension with a concentration of 1 × 10^5^ cells/mL was inoculated per well in 96-well plates. The cells were divided into four groups: (1) control group of background: cell-free culture medium group, (2) normal control group: in which only serum-free culture medium was added, (3) damaged group: in which serum-free culture medium with 2.6 mmol/L oxalate was added and incubated for 3.5 h, and (4) repair groups, including TPS0, TPS2, and TPS3 repair groups, in which different concentrations of TPS0 (10.88 kDa), TPS2 (4.82 kDa), and TPS3 (2.31 kDa), respectively, were added into the cells of damaged groups and repaired for 10 h. After the repair was completed, 10 *μ*L CCK-8 was added to each well and incubated for 1.5 h. The absorbance values were measured using the enzyme mark instrument at 450 nm to detect the repair capacity of polysaccharide.

### 2.12. Cell Morphology Observation by Hematoxylin-Eosin (HE) Staining

According to our previous study [32], changes of cell morphology were observed under an optical microscope after HE staining. 1 mL of cell suspension with a cell concentration of 1 × 10^5^ cells/mL was inoculated per well in 12-well plates and incubated for 24 h. The cells were divided into three groups: (1) normal control group: in which only serum-free culture medium was added, (2) damaged group: in which serum-free culture medium with 2.6 mmol/L oxalate was added and incubated for 3.5 h, and (3) repair groups, including TPS0, TPS2, and TPS3 repair groups, in which 80 *μ*g/mL of TPS0, TPS2, and TPS3, respectively, was added into the cells of damaged groups and repaired for 10 h. The supernatant was then removed by aspiration and the cells were washed twice with PBS. Cells were fixed with 4% paraformaldehyde for 15 min and stained with hematoxylin and eosin according to the manufacturer's instructions. Morphological changes of the cells were observed under a microscope, and the nuclei were stained in violet and cytoplasm in pink or red.

### 2.13. Changes in Reactive Oxygen Species (ROS)

The density of seeded cells and experimental grouping was the same as those in Section 2.12. The positive reagent (Rosup, 100 *μ*mol/L) of reagent kit was used as a positive control. After repair for 10 h, the cells were washed with PBS; 500 *μ*L DCFH-DA diluted with serum-free culture medium at 1 : 1000 was added and incubated for 30 min at 37°C. Then, the cells were washed 3 times with PBS to remove excess DCFH-DA. ROS distribution was observed under fluorescent microscope; the fluorescence intensity of intracellular ROS was quantitatively detected by a microplate reader.

For fluorescence quantitative detection by a microplate reader, 100 *μ*L of cells suspension with a concentration of 1 × 10^5^ cells/mL was inoculated per well in 96-well plates. After repair for 10 h, the cells were washed with PBS; then, 100 *μ*L DCFH-DA was added and incubated for 30 min at 37°C. The fluorescence intensity of intracellular ROS was quantitatively detected at 502 nm.

### 2.14. Measurement of Mitochondria Membrane Potential (Δ*ψ*m)

The density of seeded cells and experimental grouping were the same as those in Section 2.12. As a known mitochondrial membrane potential disrupter, 50 *μ*mol/L CCCP was used as a positive control. After the repair was completed, the cells were collected and centrifuged at 1000 rpm/min for 5 min. After that, the supernatant was removed by suction, and cells were rinsed twice with PBS. The Δ*ψ*m was detected according to JC-1 kit. Then the cells were stained with 200 *μ*L JC-1 dye, thoroughly mixed, and incubated in darkness at 37°C for 15 min. After treatment, the cells were detected by flow cytometer.

### 2.15. Changes in Lysosomal Integrity before and after Repair

Cell suspension with a cell concentration of 1 × 10^5^ cells/mL was inoculated per well in 12-well plates with coverslips and incubated for 24 h. The experimental grouping was the same as those in Section 2.12. The cells were washed twice with PBS and then loaded with 5 *μ*g/mL AO in DMEM for 15 min. After being repaired for 10 h, the cells were rinsed three times with PBS, and the distribution of AO in the cells was observed under fluorescence microscope.

For fluorescence quantitative detection by a microplate reader, cells (1 × 10^5^ cells/mL) were cultured in a 96-well plate (100 *μ*L/well) and were stained with AO. The red and green fluorescence were detected under enzyme mark instrument with excitation at 485 nm and emission at 530 nm (green cytoplasmic AO) and 620 nm (red lysosomal AO). Normal lysosomal integrity = (total red fluorescence intensity of normal lysosome)/(total green fluorescence intensity of normal lysosome). Lysosomal integrity = (total red fluorescence intensity)/[(total green fluorescence intensity) × (normal lysosomal integrity)].

### 2.16. Cell Cycle Assay

According to our previous study [32], changes of cell cycle progression were analyzed by flow cytometry. 2 mL of cell suspension with a cell concentration of 1 × 10^5^ cells/mL was inoculated per well in 6-well plates and incubated for 24 h. After the cells were confluent, the medium was changed to serum-free culture media and then incubated for another 12 h to achieve synchronization. The experimental grouping was the same as those in Section 2.12. After being repaired for 10 h, the cells were collected with trypsin digestion. The collected cells were washed twice with PBS and centrifuged (1000 rpm) for 5 minutes and then fixed with 70% ethanol at 4°C for 12 hours. The ethanol was removed by centrifugation (2000 rpm, 5 minutes), and the cells were washed twice with PBS. The cells were then resuspended in 200 *μ*L of propidium iodide and kept at 37°C for 15 minutes. The cell cycle was analyzed by flow cytometry to measure the amount of PI-labeled DNA in the fixed cells.

### 2.17. Changes in Apoptosis Rate before and after Repair

According to our previous study [33], apoptosis and necrosis in HK-2 cells before and after repair were measured by flow cytometer with Annexin V-FITC/PI double staining assay. The density of seeded cells and experimental grouping were the same as those in Section 2.12. The apoptosis inducer (CCCP, 50 *μ*mol/L) was used as a positive control. After the repair was completed, the cells were harvested and then stained using Annexin V-FITC/PI cell death assay kit according to the manufacturer's instructions. The cells were resuspended in 200 *μ*L of binding buffer. Then, 5 *μ*L of Annexin V-FITC was added, followed by incubation in the dark for 10 min at room temperature. The cells were resuspended in 200 *μ*L of binding buffer and stained with 5 *μ*L of PI. The prepared cells were then analyzed using a flow cytometer.

### 2.18. Statistical Analysis

The experimental data were expressed by mean ± standard deviation ($\bar{x}$ ± SD). The experimental results were analyzed statistically using SPSS 13.0 software. Multiple group comparisons were performed using one-way ANOVA, followed by the Tukey post hoc test. If *p* < 0.05, there was a significant difference; if *p* < 0.01, the difference was extremely significant; if *p* > 0.05, there was no significant difference.

## 3. Results

### 3.1. Degradation of TPS

Three degraded TPS fractions, namely, TPS1, TPS2, and TPS3, were obtained from crude TPS (TPS0) at 4%, 8%, and 14% concentrations, respectively, of H_2_O_2_. Mean Mw of TPS0, TPS1, TPS2, and TPS3 reached 10.88, 8.16, 4.82, and 2.31 kDa, respectively (Table 1). TPSs are enriched with polysaccharides.

Degradation reaction of H_2_O_2_ is moderate, and the extent of degradation can be controlled without changing the structure of the main chain of polysaccharides [34–37]. For instance, Xizhen et al. [36] performed degradation of natural soybean polysaccharide by controlling the concentration of H_2_O_2_ to obtain four polysaccharide fractions with Mw of 550, 347, 285, and 21 kDa. All degraded polysaccharide fractions had basically similar structure of the functional group. Hou et al. [37] performed degradation of *Laminaria japonica* fucoidan by changing H_2_O_2_ concentration, reaction temperature, and pH and obtained seven degraded fractions with Mw of 1.0, 3.8, 8.3, 13.2, 35.5, 64.3, and 144.5 kDa. No significant changes were observed in the major backbone structure and sulfate group content of all polysaccharide fractions.

No significant change was observed in carboxyl content of TPS before and after degradation. When concentrations of H_2_O_2_ totaled 4% and 8%, carboxyl contents of degraded TPS1 and TPS2 products reached 12.3% and 12.7%, which were slightly higher than that of TPS0 (11.2%) before degradation. The above results were attributed to the increased solubility of degraded polysaccharides (Table 1); the increase in solubility exposed numerous –COOH groups [38]. When H_2_O_2_ concentration was increased to 14%, carboxyl content of TPS3 measured 11.0% and was slightly lower than that of TPS0. This result can be explained by oxidative decarboxylation of polysaccharides induced by free oxygen atoms originating from high concentrations of H_2_O_2_ at high temperature [39].

### 3.2. Fourier-Transform Infrared (FT-IR) Spectrum of TPS

**Figure 1:**
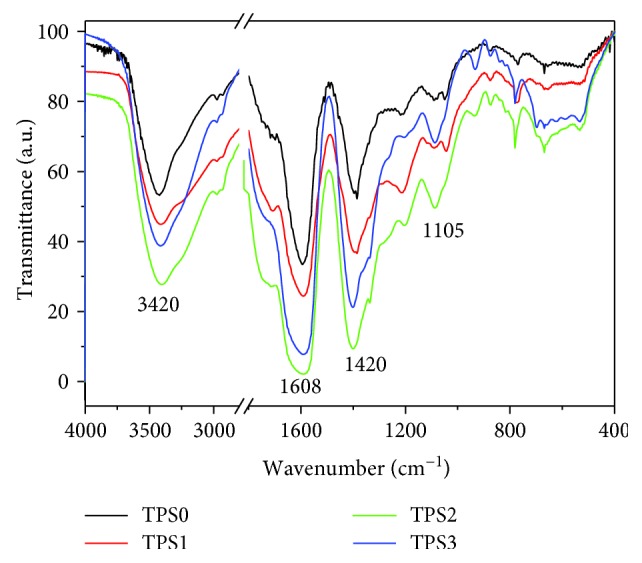
FT-IR spectra of TPSs with different molecular weights.

Figure 1 shows the FT-IR spectra of the four TPS fractions. The polysaccharide fractions presented similar spectra before and after degradation. No new peaks appeared, indicating the similar structure of the four polysaccharide fractions. The polysaccharide samples displayed strong absorption peak at 3401–3423 cm^−1^, corresponding to the stretching vibration of the hydroxyl group. Intermolecular and/or intramolecular hydrogen bonding was also observed. The absorption peak at 3000–2800 cm^−1^ was related to C–H stretching vibration. The signal at about 1608 cm^−1^ was related to C = O stretching vibration of the carboxyl group, and the signal at 1105 cm^−1^ suggested *α*-glucose pyranose ring [40].

Each polysaccharide sample manifested the same amount (2.0 mg). Thus, absorption peak intensity can reflect the content of characteristic functional groups [41]. Compared with the undegraded TPS0 fraction, absorption peaks for –OH (3412 and 3417 cm^−1^) and –COOH (1602.1 and 1610.5 cm^−1^) in degraded TPS1 and TPS2 fractions were notably stronger, respectively, indicating that TPS1 and TPS2 exposed numerous –OH and –COOH groups [38]. The absorption peak for –COOH in TPS3 weakened and showed consistency with the slightly reduced carboxylic group content (Table 2).

### 3.3. ^1^H Nuclear Magnetic Resonance (NMR) and ^13^C NMR Spectrum Analysis of TPS

As shown in FT-IR spectra, the basic structure of TPS remained undamaged during H_2_O_2_ degradation. Therefore, as representative for TPS0, ^1^H and ^13^C NMR spectra of polysaccharide were characterized, and the spectra are shown in Figure S1. The ^13^C NMR and ^1^H NMR signal assignment of TPS0 is shown in Table S1.

In ^1^H NMR spectrum, signals in the range of *δ* 4.5–5.5 ppm were assigned to the sugar ring of polysaccharides. H^−1^ proton signals derived from *α*-configuration sugar ring are detected at more than 4.95 ppm, whereas most of *β*-configuration protons will appear at less than 4.95 ppm. Thus, *β*- and *α*-configuration existed in TPS simultaneously [42]. The signals at *δ* 5.0, 3.74, 3.95, 4.25, 4.37, and 4.37 ppm corresponded to H-1 to H-6, respectively, of (1 → 4)-*α*-GalpA in TPS [43]. The signals at *δ* 4.63, 3.75, 3.56, 3.77, 3.60, and 3.91 ppm were attributed to H-1 to H-6 of (1 → 6)-*β*-Galp, whereas the signals at *δ* 5.24, 4.17, 4.09, 4.11, and 3.85 ppm were assigned to H-1 to H-5 of (1 → 2, 3, 5)-Araf, respectively [43]. The signals at *δ* 3.08, 3.10, 3.17, and 3.45 ppm were assigned to H-2, H-4, H-5, and H-6 of (1→)-*β*-D-Glcp, respectively [43]. The signals at *δ* 4.89 and 3.64 ppm were assigned to H-1 and H-6 of (1 → 4)-*α*-D-Glcp, respectively [44].

In the ^13^C NMR spectrum, the signals in the region of *δ* 100–104 ppm indicated that monosaccharides existed in the form of pyranose ring. The signals at *δ* 170–180 ppm were attributed to the uronic acid of polysaccharides [45]. The signals at *δ* 98.9, 68.2, 68.9, 77.8, 72.7, and 173.4 ppm were assigned to C-2 to C-6 of (1 → 4)-*α*-GalpA, respectively. The signals at *δ* 107.1, 73.1, 74.7, 71.5, 75.4, and 70.5 ppm corresponded to C-1 to C-6 of (1 → 6)-*β*-Galp, respectively [43]. The signals at *δ* 109.5, 81.3, 77.8, 85.3, and 70.1 ppm were assigned to C-1 to C-5 of (1 → 2,3,5)-Araf, whereas *δ* 74.3, 76.6, 70.3, 76.7, and 60.8 ppm were assigned to C-2 to C-6 of (1→)- *β*-D-Glcp, respectively [13]. The signals at *δ* 99.5 and 61.2 ppm were attributed to C-1 and C-6 of (1 → 4)-*α*-D-Glcp, respectively [44].

### 3.4. Comparison of Antioxidant Activity of TPS with Different Mw

#### 3.4.1. Hydroxyl Radical (^·^OH) Scavenging Capacity

**Figure 2:**
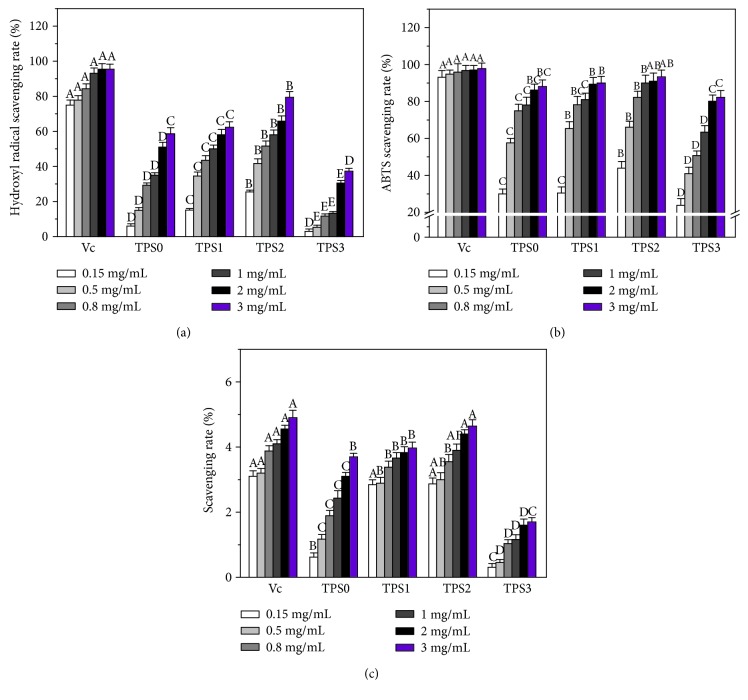
The comparison of antioxidant capacity of TPS0, TPS1, TPS2, and TPS3 at different concentrations. (a) Hydroxyl radical scavenging ability; (b) ABTS radical scavenging ability; (c) reducing power. Data were expressed as mean ± SD of five independent experiments. Different letters (A, B, C, D, E) indicate a significant difference (*p* < 0.05) between different TPSs of the same concentration.

Fenton reactions were used to investigate the ^·^OH radical scavenging ability of TPS. As shown in Figure 2(a), radical scavenging activity improved with increasing polysaccharide concentration. At the same concentration, TPS2 with midlevel Mw featured the strongest scavenging activity. For example, at the concentration of 3.0 mg/mL of TPS, ^·^OH scavenging rates of TPS0, TPS1, TPS2, and TPS3 reached 51%, 62%, 79.4%, and 37%, respectively. These values were all lower than 95.3% of Vc.

#### 3.4.2. ABTS Radical Scavenging Capacity

As shown in Figure 2(b), four TPSs showed scavenging capacity for ABTS radical in a concentration-dependent manner. At the same concentration, TPS2 exhibited the strongest scavenging ability. At 3.0 mg/mL concentration of TPS, ABTS radical scavenging rates of TPS0, TPS1, TPS2, and TPS3 totaled 88.1%, 90.0%, 93.3%, and 82.2%, respectively.

#### 3.4.3. Reducing Power

The reducing power of four TPSs followed the order TPS2 > TPS1 > TPS0 > TPS3 (Figure 2(c)). TPS2 still featured the strongest reducing power, whereas the weakest was observed for TPS3, which possessed the smallest Mw. However, reducing powers of both TPS2 and TPS3 were lower than Vc. Reducing power of the four TPSs changed concentration-dependent manner. For different polysaccharides, reducing power strengthened with increasing TPS concentration.

### 3.5. Toxicity Assessment of TPSs on Human Kidney Proximal Tubular Epithelial Cells (HK-2) Cells

**Figure 3:**
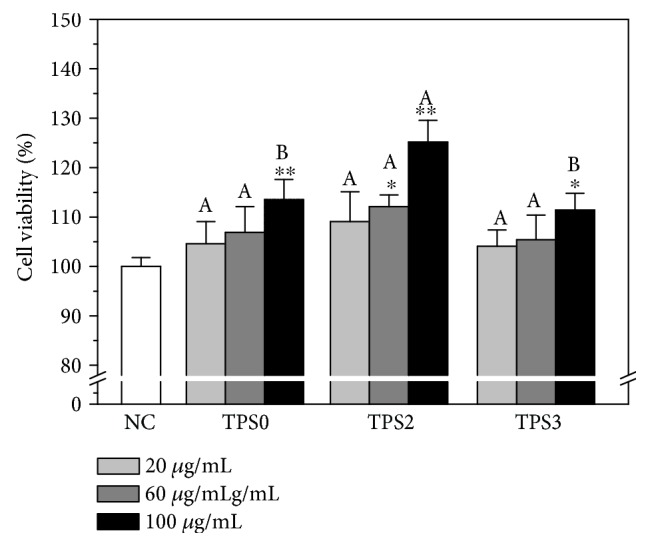
Cytotoxicity detection of TPSs with different Mw on HK-2 cells. NC: normal control. Treatment time: 24 h. Data were expressed as mean ± SD of five independent experiments. Compared with NC group, ^∗^ *p* < 0.05; ^∗∗^ *p* < 0.01. Different letters (A, B) indicate a significant difference (*p* < 0.05) between different TPSs of the same concentration.

Antioxidant activities of TPS1 (8.16 kDa) and TPS0 (10.8 kDa) showed minimal difference due to their similar Mw (Figure 2(a)–2(c)).Therefore, we only selected TPS0, TPS2, and TPS3 for the following cytotoxicity and repair experiments. As shown in Figure 3, these TPSs can promote cell proliferation within the range of 20–100 *μ*g/mL, and TPS2 exhibited the strongest promotion effect. The above results showed that these TPSs caused no cytotoxicity on HK-2 cells and promoted cell growth.

### 3.6. Repair Effect of TPS on Damaged HK-2 Cells

#### 3.6.1. Improvement of Cell Viability

The effects of oxalate concentration and injury time on the viability of HK-2 cells were shown in Figure S2. The toxicity was gradually increased with increasing oxalate concentration and exposure time. We selected the oxalate concentration of 2.6 mmol/L and the treatment time of 3.5 h for the subsequent damage experiments.

**Figure 4:**
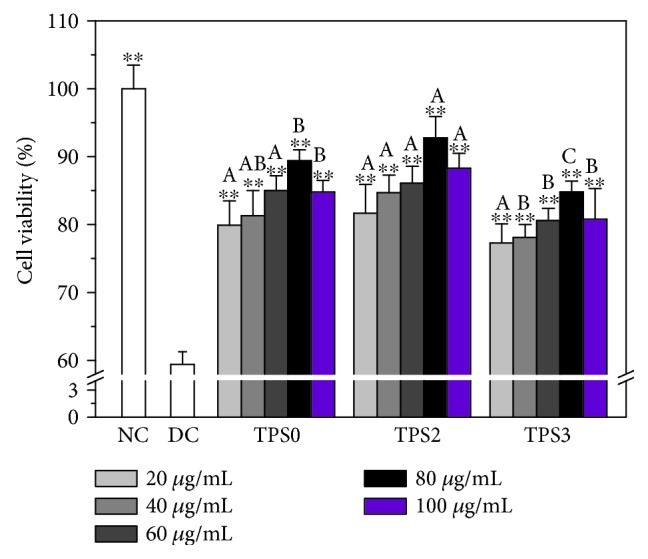
Cell viability of the damaged HK-2 cells after exposing to four TPSs with Mw. NC: normal control; DC: oxalate damaged control. Oxalate concentration: 2.6 mmol/L. Injury time: 3.5 h; repaired time: 10 h. Data were expressed as mean ± SD of five independent experiments. Compared with DC group, ^∗^ *p* < 0.05; ^∗∗^ *p* < 0.01. Different letters (A, B, C) indicate a significant difference (*p* < 0.05) between different TPSs of the same concentration.

Repair effects of TPS0, TPS2, and TPS3 on damaged HK-2 cells were compared at concentrations of 20, 40, 60, 80, and 100 *μ*g/mL (Figure 4). The best repair effect in all polysaccharides was observed at 80 *μ*g/mL concentration and decreased at concentrations higher or lower than 80 *μ*g/mL.

At the same concentration, TPS2 showed the best repair effect. For instance, cell viability of damaged HK-2 cells increased from 59.4% before repair to 89.4%, 92.8%, and 84.8% after being repaired by 80 *μ*g/mL TPS0, TPS2, and TPS3, respectively. The above results suggested that repair ability of TPSs was correlated with their Mw and is consistent with antioxidant activity.

#### 3.6.2. Repair Effect on Cell Morphology

**Figure 5:**
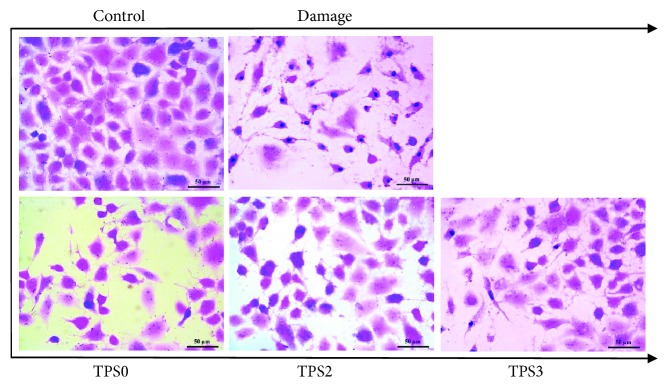
Cell morphological changes of damaged HK-2 cells after repair by TPS fractions with different Mw. Oxalate damage concentration: 2.6 mmol/L. Damaged time: 3.5 h; TPS concentration: 80 *μ*g/mL; repaired time: 10 h.

Morphological changes in damaged HK-2 cells before and after repair were observed by hematoxylin and eosin staining. As shown in Figure 5, the junctions between normal HK-2 cells were tight, and the cells were plump. When HK-2 cells were exposed to 2.6 mmol/L oxalate for 3.5 h, the cells lost their natural shape, their volume reduced, eosinophilic staining enhanced, and a large number of apoptotic cells with dense staining were formed. After repair by TPS with different Mw, cell number increased, and cell morphology was gradually restored to normal conditions. After the damaged cells were repaired by TPS2, their morphology resembled closely that of normal cells. By comparison, repair effect of TPS3 with lower Mw and TPS0 with higher Mw were weaker than that of TPS2.

#### 3.6.3. Changes in Intracellular Reactive Oxygen Species (ROS) after Repair by Different TPSs

A large amount of ROS in the body can cause oxidative damage to biological molecules (including DNA, lipids, and proteins), therefore mediating the occurrence of a series of inflammatory responses and causing cell dysfunction or death [46, 47]. The antioxidant capacity of TPS can reduce oxidative damage of cells in different degrees.

**Figure 6:**
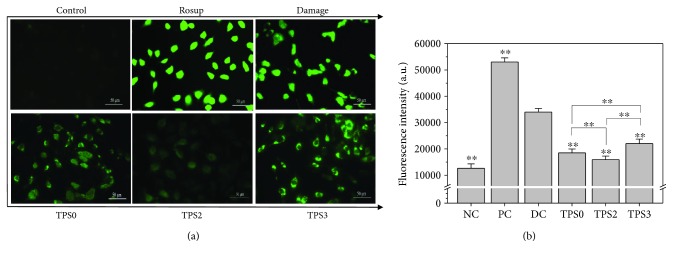
Changes in intracellular ROS of damaged HK-2 cells after being repaired by TPSs with different Mw. (a) The image of ROS distribution observed under fluorescence microscope. (b) Quantitative analysis of ROS fluorescence intensity. The green fluorescence intensity represents ROS production. NC: normal control; PC: positive control (Rosup); DC: damaged control (oxalate). TPS concentration: 80 *μ*g/mL; oxalate damage concentration: 2.6 mmol/L. Damaged time: 3.5 h; repaired time: 10 h. Data were expressed as mean ± SD of three independent experiments. Compared with DC group, ^∗^ *p* < 0.05; ^∗∗^ *p* < 0.01.

Figure 6 illustrates the intracellular ROS changes in all cell groups as detected by DCFH-DA. Following treatment of HK-2 cells with Rosup (positive control) and 2.6 mmol/L oxalate (damage control) for 3.5 h, the bright green fluorescence images were observed, indicating the high levels of intracellular ROS. After the damaged cells were repaired by TPS, fluorescent intensity displayed attenuation at different degrees, and this effect was most notable in the TPS2 treatment group (Figure 6(b)). The above results indicated that TPSs can reduce the production of intracellular ROS and alleviate oxidative damage in cells.

#### 3.6.4. Repair Effect on Mitochondrial Membrane Potential (Δ*ψ*m)

**Figure 7:**
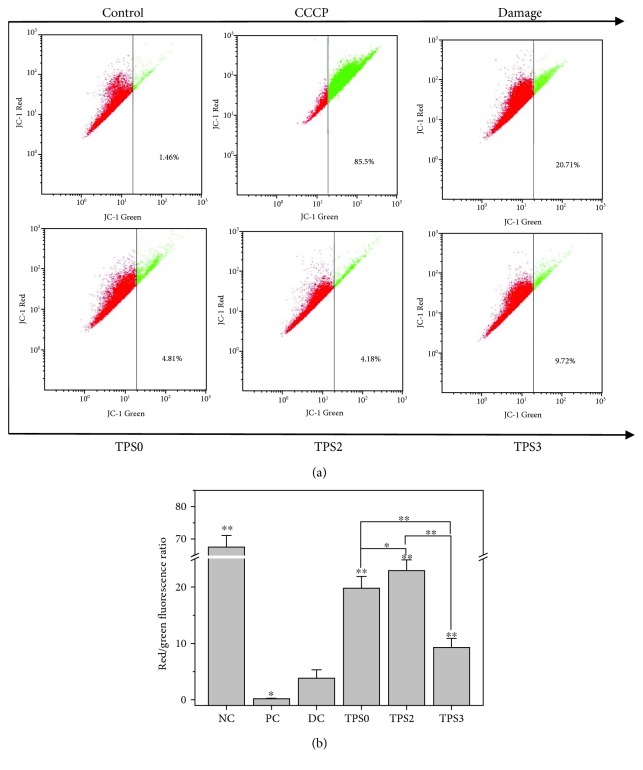
Changes in mitochondrial membrane potential of damaged HK-2 cells after being repaired by TPSs with different Mw. (a) Dot plot of Δ*ψ*m detected by flow cytometry. (b) The quantitative results of the red/green fluorescence intensity ratio. NC: normal control; PC: positive control (CCCP); DC: damaged control (oxalate). TPS concentration: 80 *μ*g/mL; oxalate damage concentration: 2.6 mmol/L. Damaged time: 3.5 h; repaired time: 10 h. Data were expressed as mean ± SD of three independent experiments. Compared with DC group, ^∗^ *p* < 0.05; ^∗∗^ *p* < 0.01.

Figure 7 shows changes in Δ*ψ*m of the damaged HK-2 cells before and after repair. The red/green fluorescence intensity ratio in the mitochondria of the normal control group reached 67.5. When cells were damaged by 2.6 mmol/L oxalate, the red/green fluorescence ratio reduced to 3.8, indicating that Δ*ψ*m was reduced evidently. However, after the damaged cells were repaired by TPS0, TPS2, and TPS3, Δ*ψ*m increased at different degrees. After the damaged cells were repaired by TPS2, the red/green fluorescence ratio in the mitochondria reached 22.9, which was higher than that of the TPS0 treatment group (19.8) and TPS3 treatment group (9.3). Thus, TPS2 showed the strongest repair effect on damaged mitochondria.

#### 3.6.5. Changes in Lysosomal Integrity before and after Repair

Acridine orange (AO), a metachromatic fluoroprobe, is a lysosomotropic component that accumulates in lysosomes by proton trapping. AO accumulation changes fluorescence emission from green in the cytoplasm to red in lysosomes [48]. Therefore, AO can be used to determine lysosomal integrity by measuring the ratio of red and green fluorescence. A low intensity of red fluorescence implies serious damage in lysosomes.

**Figure 8:**
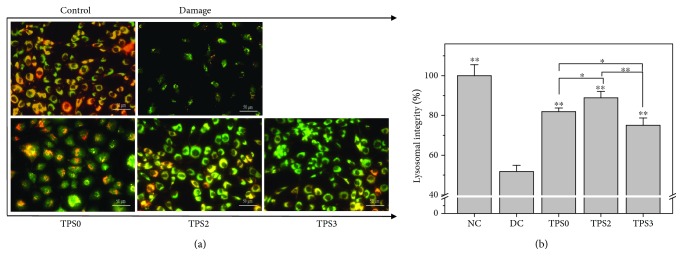
Changes in integrity of lysosomes of damaged HK-2 cells after being repaired by TPSs with different Mw. (a) The image of the integrity of lysosome observed under fluorescence microscope; (b) quantitative analysis of intracellular lysosome integrity. The cells were stained by acridine orange. TPS concentration: 80 *μ*g/mL; oxalate damage concentration: 2.6 mmol/L. Damaged time: 3.5 h; repaired time: 10 h. Data were expressed as mean ± SD of three independent experiments. Compared with DC group, ^∗^ *p* < 0.05; ^∗∗^ *p* < 0.01.

As shown in Figure 8, lysosome structure was complete (100%), and superposition of red and green fluorescence showed a strong orange-red color in normal control cells. Integrity of lysosome in damaged cells was significantly reduced (51.80%) (Figure 8(b)). However, after the damaged cells were repaired by TPS0, TPS2, and TPS3, lysosome integrity increased to 81.91%, 88.90%, and 75.03%, respectively. Therefore, TPS2 exhibited the strongest repair effect on lysosomes of cells.

#### 3.6.6. Changes in Cell Cycle before and after Repair

Cell cycle mainly includes early DNA synthetic phase (G1 phase), DNA synthetic phase (S phase), and late DNA synthetic phase (G2 phase). Arrest of the cell cycle reflects the degree of DNA damage [49].

**Figure 9:**
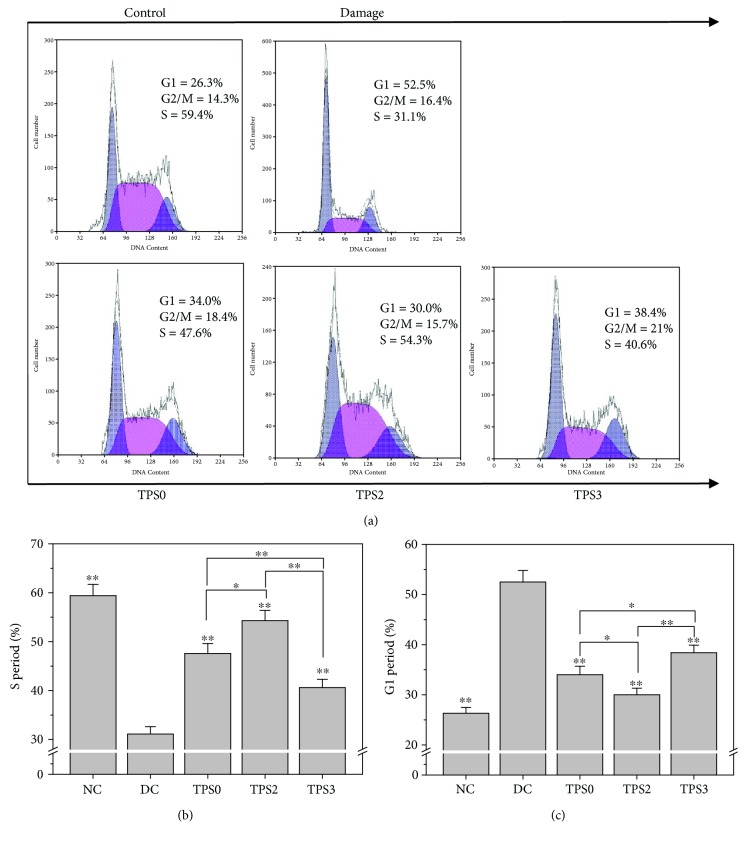
Changes in cell cycle of damaged HK-2 cells after being repaired by TPS fractions with different Mw. (a) Representative images of cell cycle; (b) the percentage of cells in the S phase; (c) the percentage of cells in the G1 phase. Oxalate damage concentration: 2.6 mmol/L. TPS concentration: 80 *μ*g/mL; Damaged time: 3.5 h; repaired time: 10 h. Data were expressed as mean ± SD of three independent experiments. Compared with DC group, ^∗^ *p* < 0.05; ^∗∗^ *p* < 0.01.

As shown in Figure 9, when normal HK-2 cells were damaged by oxalate, the percentage of cells in the S phase evidently decreased from 59.4% to 31.1% (Figure 9(b)), whereas that of cells in the G1 phase increased from 26.3% to 52.5% (Figure 9(c)). Results indicated that oxalate led to the arrest of HK-2 cells in the G1 phase. After the damaged cells were repaired by TPS0, TPS2, and TPS3, the percentage of cells arrested in the S phase increased to 40.6%–54.3%, which was higher than 31.1% observed for the damage control group. The increasing degree was related to the Mw of TPS. After the repair by TPS2, the percentage of cells in the S phase increased the most. Thus, TPS2 exhibited the strongest repair effect on DNA in damaged cells.

#### 3.6.7. Changes in Cell Apoptosis before and after Repair

We performed flow cytometric analysis to quantify apoptotic and necrotic cells using Annexin V/propidium iodide (PI) double staining. Annexin V staining was applied to reveal surface exposure of phosphatidylserine (apoptosis) and PI to reveal the loss of plasma membrane integrity (necrosis) [50, 51].

**Figure 10:**
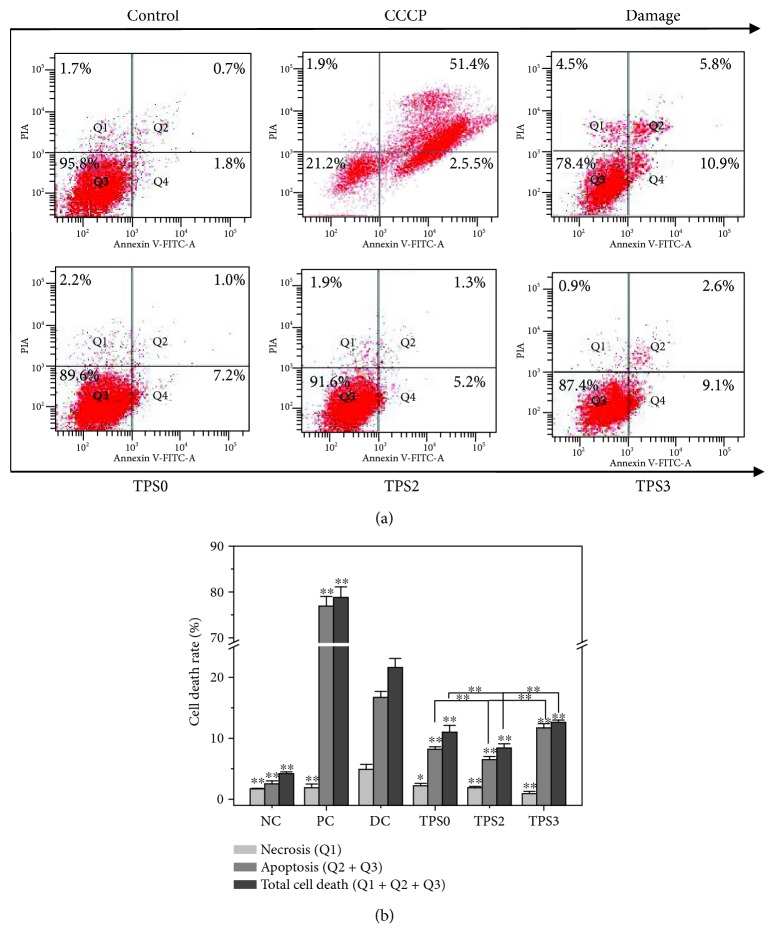
Changes in cell apoptosis and necrosis rate of damaged HK-2 cells after being repaired by TPSs with different Mw. (a) Dot plots of cell apoptosis and necrosis detected by flow cytometry. (b) Quantitative analysis of cell apoptosis and necrosis. NC: normal control; PC: positive control (CCCP); DC: damaged control (oxalate). TPS concentration: 80 *μ*g/mL; oxalate damage concentration: 2.6 mmol/L. Damaged time: 3.5 h; repaired time: 10 h. Data were expressed as mean ± SD of three independent experiments. Compared with DC group, ^∗^ *p* < 0.05; ^∗∗^ *p* < 0.01.

Figure 10(a) displays the dot plot of cellular apoptosis of the observed cells. Quadrants Q1, Q2, Q3, and Q4 denote the ratio of necrotic cells, late-stage apoptotic cells, normal cells, and early stage apoptotic cells, respectively, whereas Q2+Q4 denotes the total cell apoptotic rate. When normal HK-2 cells were damaged by oxalate, total cell apoptotic rate (Q2+Q4) increased from 2.5% to 16.7% (Figure 10(b)). After the damaged HK-2 cells were repaired by TPS0, TPS2, and TPS3, cell apoptotic rates reached 8.2%, 6.5%, and 11.7%, respectively. These rates were all lower than the 16.0% noted in the damage group. The above results indicate that TPS can reduce cell apoptosis, and TPS2 with moderate Mw exhibited the strongest repair effect.

## 4. Discussion

### 4.1. Chemical Structure Analysis of TPS

From the results of ^1^H NMR and ^13^C NMR spectrum (Figure S1), TPS comprises glucose, galactose, glucuronic acid, and arabinose. The main sugar residues included (1 → 4)-*α*-GalpA, (1 → 4)-*α*-D-Glcp, (1→)-*β*-D-Glcp, (1 → 6)-*β*-Galp, and (1 → 2,3,5)-Araf, which were consistent with the results clarified by Wang et al. [52] and Scoparo et al. [53].

FT-IR results revealed that the four polysaccharide fractions featured a similar backbone structure, but characteristic absorption peak intensities of –COOH and –OH of polysaccharide differed. This result may explain why the hydroxyl radicals produced by H_2_O_2_ degradation can attack glucosidic linkages. Oxidative scission produced diverse termini, which can be further oxidized to produce carboxyl acid. Splitting of C–C bonds within sugar residues also occurred, leading to ring-opening oxidation and formation of carboxyl groups [54]. For instance, Tian et al. [55] prepared water-soluble chitosan with low Mw (LWCS) with H_2_O_2_. FT-IR and NMR suggested no distinct change in the structures of 1,4-*β*-D-glucose main chain, whereas changes only occurred in the side chain of LWCS.

### 4.2. Effects of Mw of TPS on Antioxidant Activity In Vitro

For different types of plant polysaccharides, the best bioactivity is attributed to different ranges of Mw. For example, Xing et al. [56] reported that O_2_^·^-scavenging effect of LWCS (9 kDa) was more effective than that of high-molecular-weight chitosan (760 kDa). However, Ma et al. [57] observed that antitumor activity of high-Mw *Pleurotus eryngii* polysaccharide (413 kDa) against HepG-2 cells was better than that of low-Mw fraction (12 kDa).

#### 4.2.1. Causes of Low Antioxidant Activity and Cell Repair Ability of Low-Mw TPS

The antioxidant activity of polysaccharides is closely correlated with Mw. As shown in Figure 2, ^·^OH, ABTS scavenging rates, and reducing power of low-Mw TPS3 (2.31 kDa) were weaker than those of moderate Mw TPS2 (4.82 kDa). This phenomenon possibly caused the significant destruction in the chain structure of TPS3 (with the lowest Mw); TPS3 featured the most loose molecular structure. The number of effective hydroxyl groups capable of chelating metal ions reduced [58, 59]. Therefore, TPS3 featured the weakest free radical scavenging ability.

Many studies have also reported similar results. Sheng and Sun [58] performed degradation of *Athyrium multidentatum* (Doll.) Ching polysaccharide and obtained four polysaccharide fractions with Mw of 14,528 (CPA-1), 12,370 (CPA-2), 11,548 (CPA-3), and 6403 Da (CPA-4). At a concentration of 0.2 mg/mL, DPPH-free radical scavenging rate of CPA-1, CPA-2, CPA-3, and CPA-4 totaled 0.687, 0.605, 0.429, and 0.420, respectively. In other words, polysaccharides with low Mw show weak antioxidant activity. Lai et al. [60] extracted two Mw of mung bean polysaccharides by ethanol precipitation. At a concentration of 0.8 mg/mL, DPPH-free radical scavenging rate (70.2%) of mung bean polysaccharide with low Mw (45 kDa) was weaker than that of mung bean polysaccharide (91.6%) with high Mw (83 kDa).

#### 4.2.2. Causes of Low-Antioxidant Activity and Cell Repair Ability in High-Mw TPS

Polysaccharides can supply single electrons or hydrogen atoms to terminate free radical chain reaction and achieve radical scavenging activity [61, 62]. In comparison with low-Mw polysaccharides, high-Mw polysaccharides feature stronger winding function, a more compact structure, stronger hydrogen bond, and less exposed external active group and thus possess weaker ability to terminate free radical chain reaction.

Polysaccharide bioactivity depends on helical structure of the main chain and the presence of hydrophilic groups (hydroxyl group) located on the outside surface of the polysaccharide helix [63]. High-Mw polysaccharides possess several branched chains, large molecular volume, and steric hindrance, resulting in the easily disintegrated bioactive triple-helical polymerization structure [58, 59, 64, 65]. High-Mw of polysaccharides also exhibit limited physical properties, such as low water solubility and high viscosity, affecting their bioactivities. The repair effect in cell also reduces due to the significant increase in resistance of large volume of polysaccharide molecules into cells [66].

Many studies have reported the weak antioxidant activity of polysaccharides with high Mw [67, 68]. For example, Zha et al. [67] extracted and obtained three polysaccharide fractions from rice bran with hot-water method; the Mw ranged from 1.2 × 10^5^ Da to 6.3 × 10^5^ Da (PW1), 3.5 × 10^4^ Da to 7.4 × 10^4^ Da (PW2), and 5.3 × 10^3^ Da to 2.3 × 10^4^ Da (PW3). IC_50_ values of scavenging ABTS radical of PW1, PW2, and PW3 measured 0.35, 0.2, and 0.04 mg/mL, respectively. The above results indicate the weak antioxidant activity of high-Mw polysaccharides. Sun et al. [68] performed H_2_O_2_ degradation of k-carrageenan polysaccharide with Mw of 350,000 Da and obtained four fractions with Mw of 3.25, 5.82, 15.08, and 20.9 kDa. IC_50_ values of scavenging superoxide anion free radicals of the four degraded fractions totaled 2.65, 3.22, 6.66, and 8.13 mg/mL. As for hydroxyl radical scavenging, IC_50_ values reached 0.014, 0.049, 0.062, and 0.110 mg/mL.

#### 4.2.3. Causes of the Highest Antioxidant Activity and Cell Repair Ability in Moderate-Mw TPS

When high-Mw polysaccharides are degraded into a certain range of Mw, they can achieve optimal bioactivity. Polysaccharides with moderate Mw can not only possess sufficient spatial scale to form three-helical polymerization structure [58, 59, 64] and maintain bioactivity but also destruct highly compact molecular conformation to expose several active groups to increase hydrophilicity and stability of the structure. Steric hindrance of polysaccharides is suitable when reacting with biological receptors. Therefore, polysaccharides can exhibit strong antioxidant activity and desirable cell repair ability. TPS2 with moderate Mw can chelate with metal ions (such as Fe^2+^ and Cu^2+^) that are necessary in producing ^·^OH radicals to form complexes. Therefore, generation of radicals and initiation or progress of lipid chain reaction is inhibited [69].

Xu et al. [70] degraded crude polysaccharides from *Camellia* seed cake (COP-C) using ultrasonic wave and obtained four polysaccharide fractions, namely, COP-1, COP-2, COP-3, and COP-4, with molecular weights of 7.9, 36, 83, and 225 kDa, respectively. At the concentration of 2 mg/mL, radical scavenging capacity and reducing power order followed the order of COP-2 > COP-3 > COP-4 > COP-1. Only the polysaccharide with moderate Mw of 36–83 kDa exhibited the strongest antioxidant activity. Trombetta et al. [71] have shown that moderate Mw of polysaccharide from *Opuntia ficus-indica* (L.) cladodes also benefits enhancement of ability to repair damaged cells. The polysaccharide fraction with Mw higher than 10^4^ Da showed a wound-healing effect on damaged skin epithelium cells in mice. Wound-healing effect is more remarkable for polysaccharides with Mw ranging 10^4^–10^6^ Da than those with Mw > 10^6^ Da.

### 4.3. Polysaccharides with Strong Antioxidant Activity Feature Strong Cell Repair Ability

The four studied TPSs exhibited repair effect on damaged HK-2 cells induced by oxalate, and repair ability was positively related with antioxidant activity. TPS2, which presented the strongest antioxidant activity, also showed the strongest cell repair ability.

In living organisms, small molecules can pass through the plasma membrane directly or through the help of carrier proteins or ion channels [72], while some macromolecules, such as proteins, polynucleotides, and lipoprotein particles, are difficult to directly cross the cell membrane and needs to be transported on both sides of the cell membrane by endocytosis and efflux [73]. Sun et al. [74] confirmed that masson pine pollen polysaccharide, which has a molecular weight of 316 kDa, mainly entered RAW264.7 macrophages through receptor-regulated endocytosis rather than phagocytosis. Cobb et al. [75] indicated that polysaccharide A (PS-A) from *Bacteroides fragilis* with the molecular weight of larger than 100 kDa is endocytosed by antigen-presenting cells (APCs) and localizes to the conventional MHCII compartment (MIIC). This observation was also confirmed in primary mouse splenocytes, human THP-1 monocytes, and mouse B1 B cells. Time course studies indicated that entry and surface localization of PS-A was visible in 30 min and peaked at 6 h. Therefore, the TPS polysaccharide (2.31 ~ 10.88 kDa) used in this study can access to the HK-2 cells.

Numerous studies have shown that accumulation of ROS in vivo can attack cells and cause protein oxidation, lipid peroxidation, and nucleic acid fracture, which can affect normal cell functions and result in the occurrence of chronic diseases [46, 47, 76]. As antioxidants, polysaccharides can scavenge radicals, reduce oxidative damage of cells, and exhibit protective effects on cells [77]. For instance, *Lycium barbarum* polysaccharides can protect tissue cells from DNA damage induced by oxidative stress [78]. *Hericium erinaceus* polysaccharides can scavenge DPPH free radical, reduce ROS production, improve cell viability, inhibit reduction of mitochondrial membrane potential, and exhibit protective effects on amyloid beta-induced neurotoxicity in PC12 cells [79]. *Salvia brachyantha* extract reduces H9C2 cell apoptosis induced by *xanthine* oxidase/xanthine by preventing generation of toxic-free radicals and by enhancing the intracellular antioxidant defense system [80]. Kim et al. [81] revealed that *Psidium guajava* leaf polysaccharides can scavenge radicals to relieve H_2_O_2_-induced oxidative stress and DNA injury in Vero cells and inhibit lipid peroxidation. After cell repair by 12.5, 25, and 50 *μ*g/mL of *Psidium guajava* leaf polysaccharides, intracellular ROS production decreased from 129.5% in the damage group to 118.9%, 109.7%, and 99.7%, respectively. A polysaccharide from *Lonicera japonica* flowers remarkably reduced malondialdehyde levels, elevated superoxide dismutase and *glutathione* peroxidase activities, and protected the rat brain against ischemia/reperfusion injury [82].

**Figure 11:**
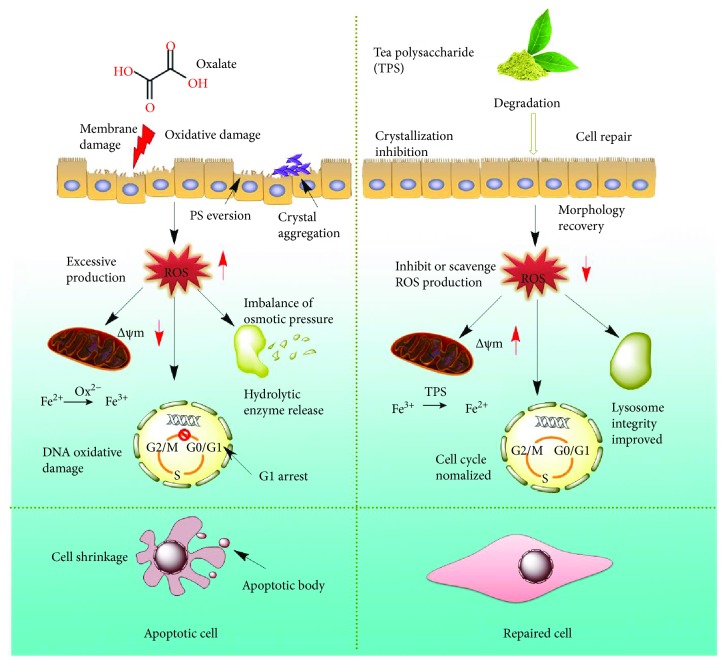
A proposed schematic illustration of the repair mechanism of damaged HK-2 cells after the treatment of TPS with different molecular weights.

On the basis of our research results, a proposed repair mechanism of damaged HK-2 cells by TPS is illustrated in Figure 11. High concentration of oxalate in urine will cause lipid peroxidation; this phenomenon leads to excessive production of ROS and damage to renal epithelial cells. TPS polysaccharides exhibit a strong ability to scavenge ROS; therefore, TPS elicited repair effect on damaged HK-2 cells. After the damaged HK-2 cells were repaired through treatment with TPS with different molecular weights, the cell viability increased, the amount of LDH released decreased, and the cell morphology was improved. When the cells were oxidatively damaged by oxalate, the permeability of the mitochondrial membrane increased, resulting in decreased Δ*ψ*m. TPS can repair the membrane potential of cells and increase Δ*ψ*m. In damaged cells, the percentage of cells in the S phase decreased but that in the G1 phase increased. After treatment with TPS, TPS promoted cell cycle progression from the G1 phase to the S phase and repaired DNA replication. Finally, TPS alleviated cell apoptosis induced by oxidative stress and decreased the underlying risk of stone formation.

## 5. Conclusions

Four TPS fractions (TPS0, TPS1, TPS2, and TPS3) with Mw of 10.88, 8.16, 4.82, and 2.31 kDa, respectively, were obtained. All TPS fractions exhibited antioxidant activity. The order of hydroxyl radical scavenging, ABTS radical scavenging activity, and reducing power was as follows: TPS2 > TPS1 > TPS0 > TPS3. The four TPSs also showed repair effects on HK-2 cells with damage induced by 2.6 mmol/L oxalate. Repair effect of TPSs was positively related with antioxidant activity. TPS2, featuring a moderate Mw, displayed the strongest antioxidant activity and cell repair ability. Compared with the damage group, cell morphology in the repaired group was closer to that of normal cells. The treated groups also yielded the following results: cell viability strengthened, mitochondrial membrane potential and integrity of lysosome improved, ROS production decreased, cells were arrested in the G1 phase, and cell apoptosis rate was reduced. All these findings indicate that these TPSs show repair effect on cell morphology, mitochondria, DNA, and other subcellular organelles in damaged HK-2 cells. Our results suggest that these TPS fractions, especially TPS2, may become potential drugs for prevention and cure of kidney stones.

## Figures and Tables

**Table 1:** Degradation conditions and physicochemical properties of TPSs with different Mw.

Polysaccharide abbreviation	H_2_O_2_ concentration C_(H2O2)_/%	Intrinsic viscosity [*η*]/mL/g	Mean molecular weights Mr/kDa	–COOH content/%	Solubility (25°C) g/100 g
TPS0	0	3.952 ± 0.130	10.88 ± 0.73	11.2	10.0
TPS1	4	3.434 ± 0.086	8.16 ± 0.42	12.3	12.5
TPS2	8	2.653 ± 0.072	4.82 ± 0.27	12.7	25.0
TPS3	14	1.842 ± 0.188	2.31 ± 0.48	11.0	33.3

**Table 2:** FT-IR characteristic absorption peak of original and degraded TPS.

Polysaccharide abbreviation	Mean molecular weights M_r_/kDa	–COOH content/%	Relative intensity of -COOH absorption peak	Functional groups characteristic absorption peak		
				-OH	-COOH	Sugar ring
TPS0	10.88	11.2	1.3	3423	1609.9	1391.1, 1142.3, 1093.9, 765.9
TPS1	8.12	12.3	2.0	3417	1602.1	1385.4, 1213.4, 778.6
TPS2	4.82	12.7	2.7	3412	1610.5	1399.2, 1087.5, 780.6
TPS3	2.31	11.0	1	3401	1608.1	1401.4, 1087.3, 780.4

[_∗_] (100-T _TPS0_): (100-T_TPS1_): (100-T_TPS2_): (100-T_TPS3_), where T represents the light transmittance.

## Data Availability

All the data supporting the results were shown in the paper and can be applicable from the corresponding author.

## Supplementary Materials

Supplementary Materials The ^13^C NMR and ^1^H NMR attribution and spectra of TPS0 used for structural elucidation are presented in Table S1 and Figure S1. The effects of oxalate concentration and injury time on the viability of HK-2 cells are presented in Figure S2.
