# Supplementary material for: Antioxidant Activities and Repair Effects on Oxidatively Damaged HK-2 Cells of Tea Polysaccharides with Different Molecular Weights
Source: Oxid Med Cell Longev. 2018 Nov 21;2018:5297539. doi: 10.1155/2018/5297539 (PMC6280578; doi:10.1155/2018/5297539)
Supplement: Supplementary Materials — The 13C NMR and 1H NMR attribution and spectra of TPS0 used for structural elucidation are presented in Table S1 and Figure S1. The effects of oxalate concentration and injury time on the viability of HK-2 cells are presented in Figure S2. [file 5297539.f1.doc]

Supporting information

Table S1. The ^13^C NMR and ^1^H NMR attribution of TPS0.

| Composition* | Chemical shifts (δ ppm) | | | | | |
| --- | --- | --- | --- | --- | --- | --- |
|  | C-1/H-1 | C-2/H-2 | C-3/H-3 | C-4/H-4 | C-5/H-5 | C-6/H-6 |
| (1→2,3,5)-Araf | 109.5/5.24 | 81.3/4.17 | 77.8/4.09 | 85.3/4.11 | 70.1/3.85 |  |
| (1→4)-α-GalpA | 98.9/5.00 | 68.2/3.74 | 68.9/3.95 | 77.8/4.25 | 72.7/4.37 | 173.4/4.37 |
| (1→4)-α-D-Glcp | 99.5/4.89 |  |  |  |  | 61.2/3.64 |
| (1→)- β-D-Glcp |  | 74.3/3.08 | 76.6/- | 70.3/3.10 | 76.7/3.17 | 60.8/3.45 |
| (1→6) -β-Galp | 107.1/4.63 | 73.1/3.75 | 74.7/3.56 | 71.5/3.77 | 75.4/3.60 | 70.5/3.91 |

[*] Galp: [galactose](http://dict.youdao.com/w/galactose/); Glcp: glucose; Araf: Arabinose; GalA: glucuronic acid

Fig. S1. NMR spectra of TPS0. (A) ^1^H NMR spectrum; (B) ^13^C NMR spectrum.

Fig. S2. Effects of oxalate concentration (A) and injury time (B) on the viability of HK-2 cells.
